# The Radical Treatment of Local Venereal Diseases by Cauterization and Circumcision

**Published:** 1919-01

**Authors:** 


***The Radical Treatment of Local Venereal Diseases by Cauterization and Circumcision.*** By Major L. QUALLS. *The Military Surgeon,* August, 1918.

The treatment of venereal diseases by cauterization and circumcision may be summarized as follows :

Thorough cleansing of the field of operation with iodine; cauterization of all visible ulcerations, of ulcerated areas on glans and prepuce ; and circumcision.

In cases of gonorrheal phimosis, where a chancroidal infection is found after dorsal slit, actual cautery is recommended.

In cases where the infection is so extensive that circumcision is impossible, owing to lack of healthy tissues, the entire foreskin is burned off.

In cases where untreated phagedenic chancroids have destroyed the glans penis, amputation is carried out by burning through the skin, connective and penile tissues, procuring the urethra with forceps and fixing it with sutures. Antiseptic powder is then applied and the wound dressed in 5 to 6 days' time.

In cases of suspected primary syphilis, the local lesion is excised, the area of excision cauterized and the excised ulcer sent to the laboratory, where a small amount of clear serum is expressed for dark field examination.

The apparatus required for cauterization is a Primus kerosene stove and 2 or 3 Mayo cautery irons; the only instruments needed after cauterization are hemostats, scissors, forceps, needles and knife.

The author gives in detail a series of cases treated by the above described method. In 54 cases with gonorrheal urethritis, primary or secondary syphilis and buboes, including 2 circumcisions and 2 amputations by cauterization, the average time spent in hospital was 16.8 days. The clinical records of 6 cases of chancroidal infection with phimosis, treated by cauterization and circumcision, show an average of 10.7 days in hospital, while 8 cases of infection without phimosis or paraphimosis, required an average of 9 days of hospital treatment.

The treatment by cauterization and circumcision has many advantages over the older methods:

(1) It shortens hospital treatment, simplifies after treatment, decreases morbidity and pain.

(2) It decreases the number and extent of complications.

(3) It decreases deformity and mutilation and aids in the early diagnosis of syphilis.

(4) Cauterization arrests instantly the spread of phagedena.

Its only disadvantage is that, in most cases, a general anesthetic is required.

